# Impaired attention and cognitive deficits associated with pain and autonomic symptoms in hypermobile Ehlers-Danlos syndrome: a pilot study

**DOI:** 10.1007/s10286-026-01191-7

**Published:** 2026-03-09

**Authors:** Katharina Müller, Joana C. Thiel, Lena Schopen, Bruno Fimm, Jörg B. Schulz, Andrea Maier

**Affiliations:** 1 Department of Neurology, Medical Faculty RWTH Aachen University, Pauwelsstraße 30, 52074 Aachen, Germany; 2 JARA-BRAIN Institute Molecular Neuroscience and Neuroimaging, Forschungszentrum Jülich GmbH and Medical Faculty, RWTH Aachen University, Aachen, Germany; 3 Clinic for Gynaecology and Obstetrics, Medical Faculty, RWTH Aachen University, Aachen, Germany; 4 Developmental Psychology and Research Methods Teaching and Research Area, Institute of Psychology, RWTH Aachen University, Aachen, Germany

**Keywords:** Hypermobile Ehlers-Danlos syndrome (hEDS), Postural orthostatic tachycardia syndrome (POTS), Cognitive impairment, Executive function, Coordination, Proprioception

## Abstract

**Purpose:**

Patients with hypermobile Ehlers-Danlos syndrome (hEDS) frequently present with circulatory dysfunction, including postural orthostatic tachycardia syndrome (POTS), and cognitive impairments, leading to substantial disability and limitations in daily functioning. Few studies have examined attention, and concentration and associated conditions in hEDS. In this case–control study, we used a comprehensive cognitive test battery to assess whether cognitive performance is impaired in individuals with hEDS and depends on different body positions.

**Methods:**

Twenty-nine patients and 29 healthy controls (HC) were enrolled. Baseline cognitive assessments included the Performance Scale of an intelligence test (LPS), Montreal Cognitive Assessment (cognitive deficit screening), and Test of Attentional Performance (TAP). The main cognitive tests for assessing the effects of body position were conducted in randomized conditions (supine, standing, and standing legs-crossed) and included the Stroop test, Corsi block-tapping test, Trail Making Test Part B, and Wechsler Memory Scale-revised.

**Results:**

Compared to HC, patients with hEDS had higher intellectual performance (*p* < .050), but besides relevant comorbid conditions, also significantly impaired attention in the TAP (*p* < .010) and an impairment of executive function assessed by the Stroop test (*p* < .010) in the legs-crossed compared to the supine position.

**Conclusion:**

Attention in the hEDS group was impaired compared to HC, and executive performance was dependent on body position. Individuals performed worse when standing legs-crossed. Hence, impaired proprioception as present in hEDS may, along with comorbid conditions such as pain, be a contributing factor affecting executive function.

## Introduction

Ehlers-Danlos syndromes (EDS) are rare, congenital disorders comprising a heterogeneous group of hereditary connective tissue disorders. Among the 13 recognized subtypes, the hypermobile type (hEDS) is the most common subtype and mostly affects women. hEDS is characterized by hypermobility of the joints, mild hyperextensibility of the skin, and fragility of the tissues [1–3]. Muscles, ligaments, joints, internal organs, and blood vessels may be affected in this condition [1]. Besides typical symptoms of joint dislocations and pain, patients with hEDS often express difficulties in concentration. However, there is limited data on the prevalence and pathophysiology of these cognitive issues [4–6]. One previous study in 28 patients with hypermobility spectrum disorder/hEDS showed cognitive impairments in patients with hEDS in terms of visuospatial problem-solving, attention, and memory compared to healthy controls (HC) [4]. Possible mechanisms of cognitive impairment include pain [7–9] and orthostatic intolerance (OI) [10]. While up to 90% of patients with postural orthostatic tachycardia syndrome (POTS) report cognitive complaints [11], only a few studies showed cognitive impairments. These impairments in patients with POTS mainly comprise attention, working and short-term memory, as well as executive functioning, and were found to be position dependent with worsening in the upright position [12–16]. Up 80% of patients with hEDS present with syndromes of OI, such as orthostatic hypotension (OH) or POTS [17–19].

Recent evidence supports a multifactorial model of cognitive impairment in hEDS. A retrospective cohort demonstrated that 79% of patients with hEDS exhibited reduced orthostatic cerebral blood flow velocity and frequent autonomic dysfunction [20], suggesting impaired cerebral perfusion as a potential mechanism. Small fiber neuropathy (SFN) and thus pain is a frequent symptom in 60% of patients with hEDS, indicating peripheral and autonomic nerve involvement [21, 22]. Furthermore, others found significant correlations between OI symptoms and cognitive complaints, supporting the view that dysautonomia contributes to cognitive dysfunction in this population [23]. Although the precise mechanisms remain unclear, both distraction and interruption may be contributors to impaired cognition: pain may divert attentional resources and engage overlapping neural networks involved in cognitive control [24], whereas OI may transiently reduce cerebral perfusion, causing temporary lapses in attention and executive function. Recent evidence highlights the complex interplay between subjective and objective cognitive performance in hypermobile hEDS [24]. While objective cognitive functioning is largely preserved, patients frequently report fluctuating cognitive difficulties influenced by pain, fatigue, and mood. A study published in 2025 specifically examined both objective and subjective cognitive performance in patients with hEDS, capturing fluctuations over time. It found that objective cognitive functioning was largely preserved, whereas reported cognitive problems were mostly subjective and varied, influenced by pain, fatigue, and depression [25]. Changes in posture, such as standing, might further affect cognition by altering cerebral perfusion due to autonomic dysregulation. Assessing cognitive performance in both supine and upright positions might allow identification of orthostasis-related deficits in attention or processing speed. Moreover, compensatory maneuvers such as leg-crossing, which increase venous return and cerebral blood flow, could help mitigate orthostatic and cognitive symptoms [26, 27].

Thus, we investigated whether patients with hEDS have impaired cognitive function compared to HC, whether cognitive function is influenced by comorbid conditions, e.g., pain, OI, autonomic complaints, or psychological distress, and whether cognitive performance is influenced by different body positions.

## Materials and methods

### Screening process and study design

A priori power analysis was performed to estimate the required sample size. On the basis of this analysis, approximately 30 participants per group were deemed sufficient to detect a medium-sized effect with adequate statistical power of 0.95. This approach was considered appropriate during study planning, as detecting smaller effects would have required a substantially larger sample size.

**Fig. 1:**
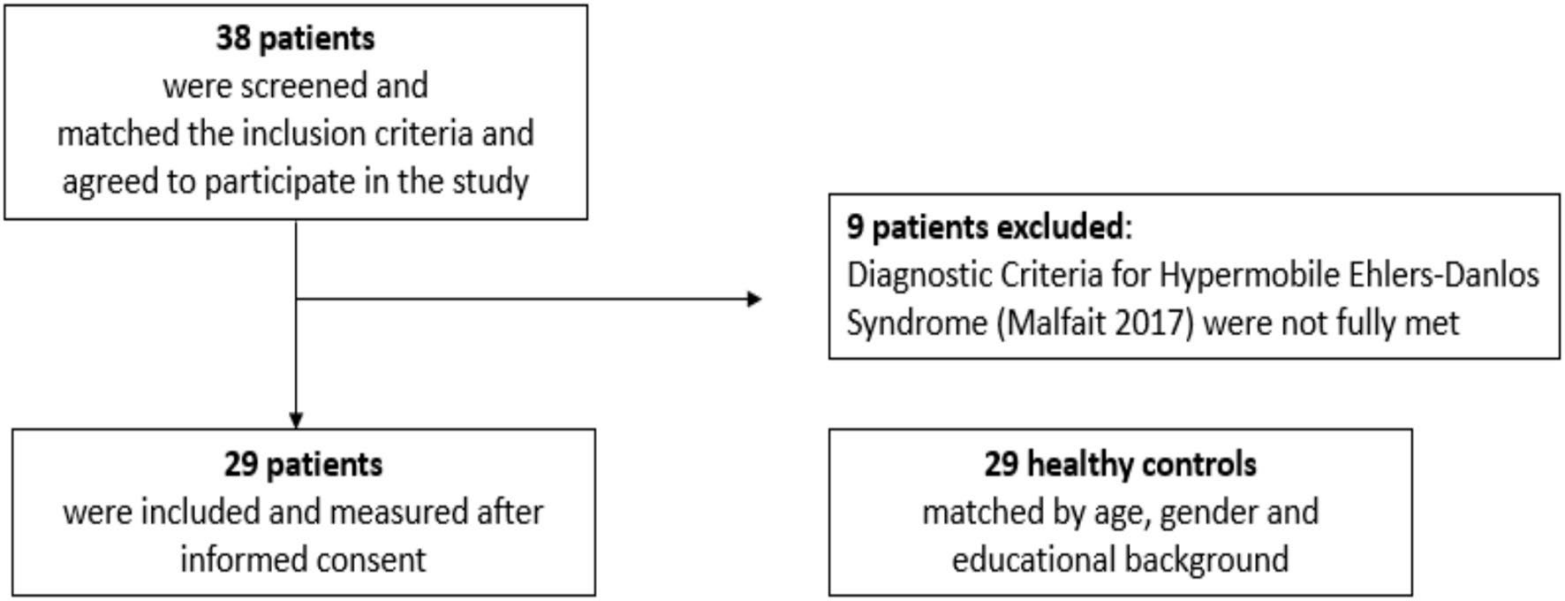
Flow diagram of the screening process

Twenty-nine patients with hEDS and 29 HC, matched by age, gender, and educational background, were prospectively enrolled in the present pilot study between 2017 and 2020 (Fig. 1). The study was performed in compliance with relevant laws, was approved by the institution’s ethics committee (EC 031/17), and conducted in accordance with the Declaration of Helsinki (registered as NCT03681080). Every participant provided written informed consent prior to participating in the study.

hEDS was clinically diagnosed by an expert on the field based on the current gold standard of diagnostic criteria for hEDS [2]. Exclusion criteria included pregnancy, implanted pacemaker or deep brain stimulation, drug abuse, and severe heart failure. Additionally, for HC, exclusion criteria comprised the use of antihypertensive medication, previous neurological or psychiatric illness, as well as previous symptoms of dizziness, history of syncope, and known iron deficiency. A detailed medical history including orthostatic history was obtained for all participants. A physical examination and assessment of the Beighton score was only undertaken on the patients. The participants were asked not to take their medication on the day of the examination. A longer break from medications that could affect cognitive abilities (e.g., stimulants, serotonin and norepinephrine reuptake inhibitors [SNRIs]) was not required, as the patients were dependent on them. For the tilt table, fasting was also required (no breakfast, no caffeine). Circulatory disorders were tested using the standardized tilt-table examination, as previously described [13]. POTS was defined according to the international guidelines as a persisting increase in heart rate (HR) of at least 30 beats/min within 10 min of orthostasis, in the absence of OH, and associated with orthostatic symptoms including palpitations, dizziness, headache, and presyncope for at least 3 months [28, 29]. OH was defined by a significant decrease in the systolic blood pressure (a drop of at least 20 mmHg) or diastolic blood pressure (a drop of at least 10 mmHg) within 3 min after tilting [28, 29].

### Questionnaires

General physical and mental health was assessed by the Epworth Sleepiness Scale (ESS) [30, 31], the RAND 36-Item Health Survey [32], which was analyzed according to the German manual [33], the German version of the Composite Autonomic Symptom Score (COMPASS-31) [34, 35], the Beck Depression Inventory II (BDI II) [36], the Beck Anxiety Inventory (BAI) [37], a pain questionnaire (painDETECT) [38], and a scale that assesses symptoms of OI from lying compared to standing or prolonged standing (Winker Scale); the latter comprises ten questions rated from zero to four based on symptom frequency with a total score of 40 points [39].

### Baseline cognition

At cognition baseline, we evaluated the educational level (≤ 12 years vs. > 12 years of education), administered the Performance Scale of an intelligence test, the Leistungsprüfungssystem (LPS, intelligence test) [40], the Montreal cognitive Assessment (MoCA; screening for cognitive deficits) [41], and the Test Battery for Attention (TAP; attention capacity) [42].

### Main cognition

Cognitive performance during different body positions was assessed using the Stroop test (selective attention) [43], the Corsi block-tapping test (spatial short-term memory and working memory) [44], the Trail Making Test Part B forward and backwards (TMT-B; visual attention and task switching) [45], and the Wechsler Memory Scale-R (memory functions) [46].

**Fig. 2:**
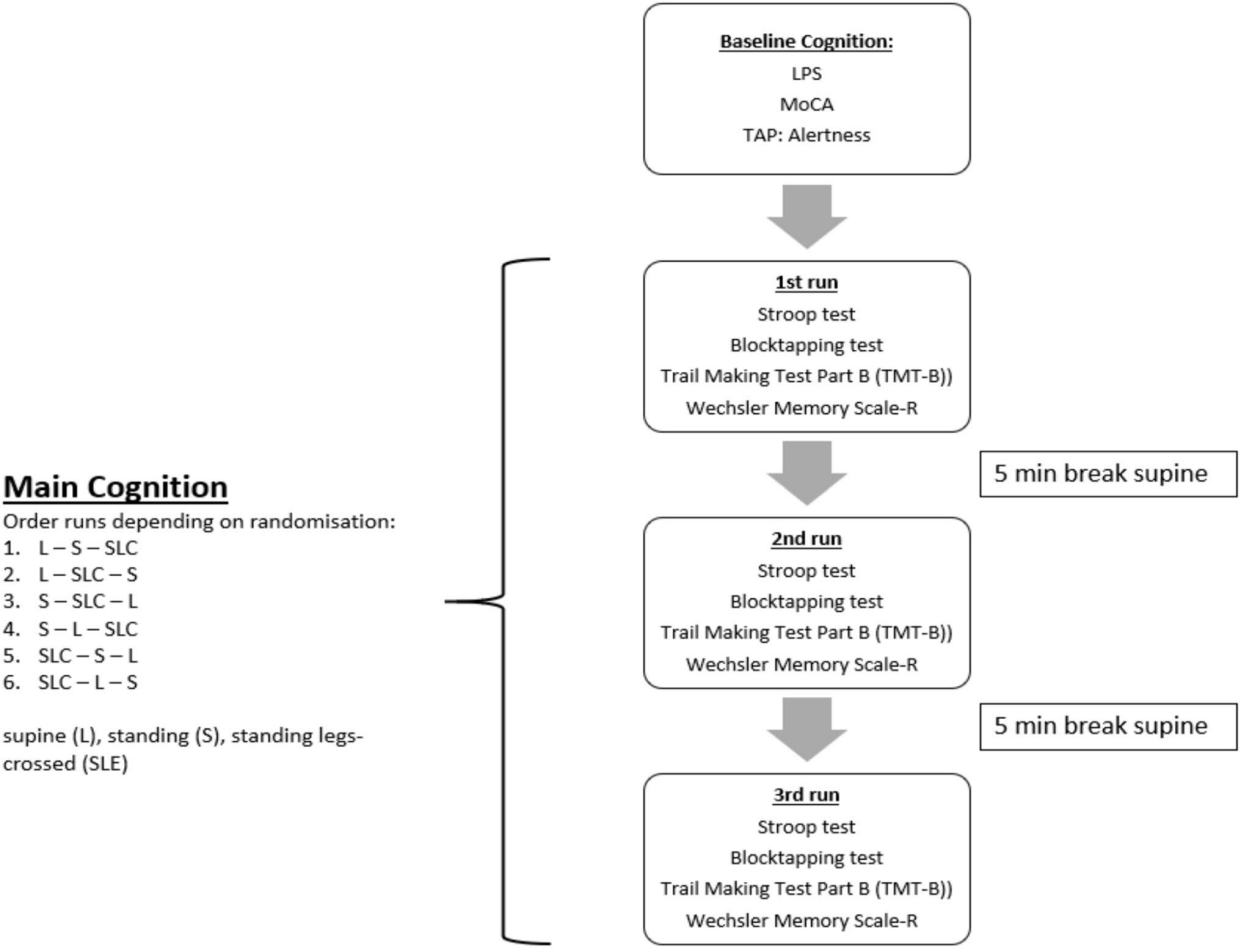
Study protocol

All tests were performed in three runs with varying body positions: supine (L), standing (S), and standing with legs-crossed (SLC). The order of the positions was randomized between participants to counteract any training effects. The tests followed a standardized procedure with 5-min breaks in a supine position between each run to mitigate fatigue effects (Fig. 2). Different versions of the Stroop test were available, and we used them accordingly to minimize learning effects. For the other tests, parallel forms were not available, but body positions were randomized to further reduce potential practice effects. In the Stroop test, participants must quickly name the ink color of a word, which may conflict with the word’s meaning. Reaction time from the start to the completion of the task was recorded, with faster responses indicating better selective attention and cognitive control. The detailed procedure was described previously [13].

### Statistical analysis

Statistical analysis was performed with SPSS (version 28.0). The primary endpoints were the results of the cognitive tests (TMT-A, TMT-B, and Stroop test) measured as changes in performance between lying, standing, and cross-legged positions. To account for multiple comparisons, the alpha level was adjusted using the Bonferroni correction. Accordingly,* p* values < 0.0125 were regarded as statistically significant. Secondary endpoints included hemodynamic changes between different body positions. Specifically, we assessed blood pressure change (mmHg) and heart rate change (beats per minute) between lying, standing, and cross-legged positions.

Differences between HC and hEDS in normally distributed metric data were assessed using an unpaired *t* test. Potential differences in education, as categorical data, were assessed by the chi-square test. Post hoc *t* tests, investigating the significant interaction between position and group on reading time in the Stroop test, were performed. Age-based normative T-values were used for the analysis of the Stroop test. A T-score of 50 represents average performance for the respective age group. Scores above 50 indicate better-than-average performance (e.g., 60 corresponds to one standard deviation above average), while scores below 50 indicate worse-than-average performance (e.g., 40 corresponds to one standard deviation below average). A repeated-measured analysis of variance (ANOVA) was used to determine the effect of positional differences on cognitive performance. We determined the group (hEDS vs HC) as *between factor* and the position (L vs S vs SLC) as *within factor*. In addition, the analysis was performed using OI as a covariate. Pearson’s correlation analysis was performed to assess a linear relationship between the cognition tests, pain, anxiety, and depression. Results are reported as mean ± standard deviation (SD).

## Results

### Demographics and questionnaires

Demographic characteristics are shown in Table 1. The majority (83%) had a school education of more than 12 years. Sixteen participants (55%, 15 female, mean age 32 years) were diagnosed with OI as POTS (*n* = 9, 8 female, mean age 28 years) or OH (*n* = 7, all female, mean age 36 years). Sixteen out of 29 patients with hEDS (55%) were diagnosed with small fiber neuropathy (SFN), a prevalence comparable to that reported in other studies [21, 22]. Significant differences between hEDS and HC were observed in the self-assessment questionnaires. In contrast to HCs, the patients with hEDS reported significantly more orthostatic complaints during standing (Winker Scale EDS 27.31 ± 1.49 vs. HC 2.69 ± 0.63, *p* < 0.001). Although the HC had rather high COMPASS-31 score, autonomic symptom burden in hEDS was significantly higher than in HC (EDS 57.314 ± 2.51 vs. HC 15.97 ± 2.48, *p* < 0.001) and patients with hEDS experienced higher levels of pain (EDS 4.21 ± 2.077 vs. HC 0.54 ± 1.290, *p* < 0.001). In hEDS, there were a higher level of anxiety (BAI, EDS 19.14 ± 2.34 vs. HC 3.45 ± 0.4, *p* < 0.001) and more depressive symptoms (BDI, EDS 13.72 ± 1.92 vs. HC 6.14 ± 1.25; *p* = 0.002). Patients with hEDS had higher levels of daytime sleepiness (EDS 10.41 ± 1.24 vs. HC 6.79 ± 0.69; *p* = 0.014). Mental and physical health was significantly reduced in hEDS compared to HC in all subscales of the RAND 36-Item Health Survey, with exception of emotional role function and psychological well-being (Table 2).

**Table 1:** Demographic data and autonomic evaluation of patients with hEDS compared to healthy controls (HC)

Parameter	hEDS	HC	*p* value
	*N* = 29	*N* = 29	
Age, years	36.72 ± 1.85	35.90 ± 2.02	.764
Education (> 12 years)	24 (0.83 ± 0.07)	23 (0.79 ± 0.08)	1.000
Sex (female)	28 (0.97 ± 0.03)	28 (0.97 ± 0.03)	1.000
POTS	9	0	< .001***
OH	7	0	< .001***
SFN	16	0	< .001***
Beighton score	7.10 ± 0.31	0	< .001***

Data are presented as the frequency and/or mean ± standard deviation

*POTS* postural orthostatic tachycardia syndrome,* OH* orthostatic hypotension, SFN small fiber neuropathy

*** p < .001

**Table 2:** Comparison of the clinical data of the preliminary studies of patients with hEDS compared to healthy controls (HC)

Parameter	hEDS	HC	*p* value
Preliminary studies	*N* = 29	*N* = 29	
Winker Scale	27.31 ± 1.49	2.69 ± 0.63	< .001***
ESS	10.41 ± 1.24	6.79 ± 0.69	.014*
COMPASS-31	57.31 ± 2.51	15.97 ± 2.48	< .001 ***
BDI	13.72 ± 1.92	6.14 ± 1.25	.002**
BAI	19.14 ± 2.34	3.45 ± 0.84	< .001***
Pain questionnaire (NRS)	4.21 ± 2.077 (1 NT)	0.54 ± 1.290 (1 NT)	< .001***
RAND 36: Physical function	40.69 ± 3.65	88.97 ± 3.19	< .001***
RAND 36: Physical role function	5.17 ± 2.28	87.93 ± 5.49	< .001***
RAND 36: Physical pain	24.93 ± 3.86	91.79 ± 3.45	< .001***
RAND 36: General health	20.79 ± 2.24	75.83 ± 3.92	< .001***
RAND 36: Vitality	24.31 ± 3.41	57.76 ± 3.63	< .001***
RAND 36: Social functional capacity	40.52 ± 4.75	90.52 ± 3.91	< .001***
RAND 36: Emotional role function	75.86 ± 7.19	81.60 ± 5.28	.525
RAND 36: Psychological well-being	64.14 ± 4.24	76.41 ± 3.07	.023**
LPS	51.38 ± 0.82	46.68 ± 1.86 (1 NT)	.026*
MOCA	27.76 ± 0.36	28.79 ± 0.20 (1 NT)	.098
TAP median	38.90 ± 1.37	44.5 ± 0.34 (1 NT)	.006**

Data are presented as the frequency and/or mean ± standard deviation

*ESS* Epworth Sleepiness Scale,* COMPASS-31* German version of the Composite Autonomic Symptom Score,* BDI II* Beck Depression Inventory II,* BAI* Beck Anxiety Inventory,* painDETECT* pain questionnaire,* NRS* Numeric Rating Scale,* RAND 36* German version of the RAND 36-Item Health Survey,* LPS* Leistungsprüfungssystem (intelligence test),* MoCA* Montreal cognitive Assessment,* TAP* Test Battery for Attention,* NT* not tested (i.e., there were participants who were either not tested in a test or forgot to fill out a questionnaire)

*p < .05; **p < .01; ***p < .001

### Baseline cognition

**Fig. 3:**
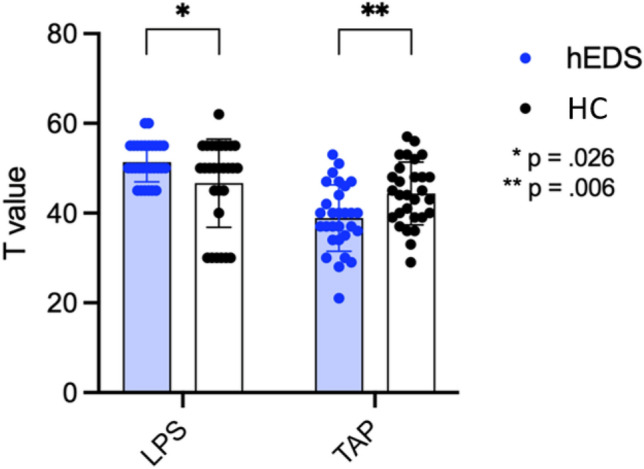
Comparison of results in LPS and TAP between patients with hEDS and healthy controls (HC). *p < .05; **p < .01

Concerning cognition baseline, patients with hEDS had significantly lower reaction times in the TAP (38.9 ± 1.36; *p* < 0.010) compared to HC (44.5 ± 0.33), but in LPS, hEDS performed better (51.38 ± 0.81) than HC (46.68 ± 1.85; *p* < 0.050) (Fig. 3) These results highlighted lower attention capacity in patients with hEDS, although their intelligence was higher.

### Main cognition

**Fig. 4:**
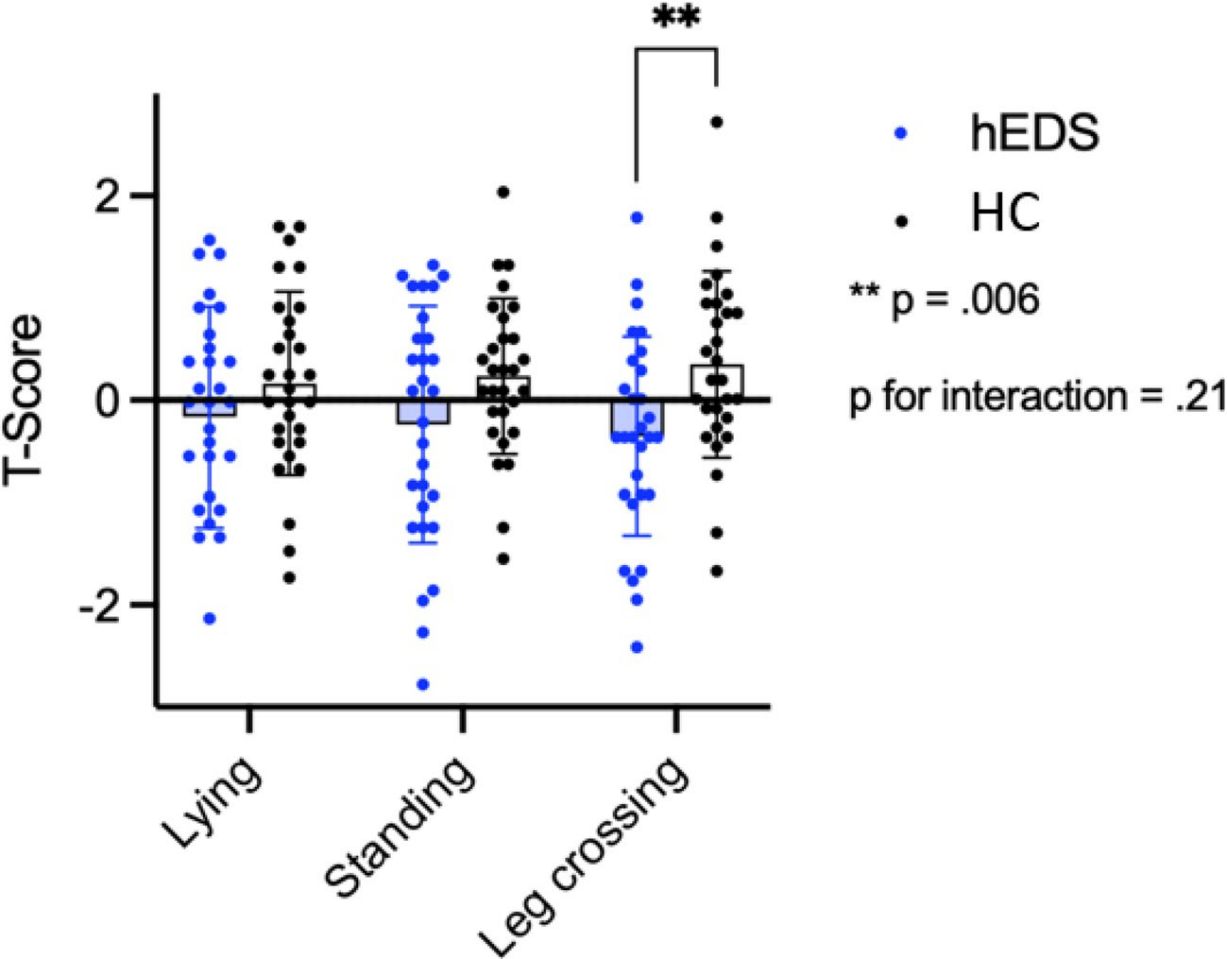
Results of Stroop test in the different positions. In comparison hEDS vs. HC. *p < .05; **p < .01

When age-standardized T-scores were compared, patients with hEDS showed significantly lower values in the Stroop test during the standing legs-crossed condition compared to HC (hEDS 54.1 ± 1.93 vs. HC 61.6 ± 1.81; *p* < 0.010). Since higher T-scores indicate better performance, this result reflects impaired executive function in the hEDS group during this specific challenge (Table 3, Fig. 4). The robust ANOVA showed no influence of the position in the whole study group (HC and hEDS). There was a significant interaction between groups and positions [*F*(2, 112) = 3.221, *p* < 0.044], indicating that the executive functioning measured by the Stroop test was influenced by position only in patients with hEDS (Fig. 4). Further, the post hoc tests revealed a significant lower performance during the Stroop test in the legs-crossed position compared to the supine position in patients with hEDS only (Table 4). After controlling for the condition of OI (patients with POTS or OH), the position effect did not remain significant [*F*(2, 110) = 1642, *p* < 0.198].

**Table 3:** Test results of the main examination of the patients with hEDS compared with the healthy controls

Main investigation	hEDS	HC	*p* value
	*N* = 29	*N* = 29	
Stroop test (L)	56.90 ± 1.52	59.38 ± 1.263	.215
Stroop test (S)	54.83 ± 2.096	59.41 ± 1.383	.074
Stroop test (SLC)	54.10 ± 1.936	61.62 ± 1.816	.006**
Corsi block-tapping test (L)	5.24 ± 0.183	5.31 ± 0.223	.812
Corsi block-tapping test (S)	5.07 ± 0.192	5.28 ± 0.185	.441
Corsi block-tapping test (SLC)	5.03 ± 0.23	5.48 ± 0.190	.138
TMTB (L)	44.72 ± 1.139	46.69 ± 1.178	.235
TMTB (S)	43.66 ± 1.375	46.76 ± 1.266	.102
TMTB (SLC)	45.00 ± 1.081	48.24 ± 1.125	.042*
WMS-R (L)-(f)	51.72 ± 1.189	47.38 ± 1.906	.105
WMS-R (L)-(b)	46.55 ± 1.424	46.21 ± 2.064	.891
WMS-R (S)-(f)	49.45 ± 1.982	46.48 ± 1.849	.279
WMS-R (S)-(b)	46.38 ± 1.756	46.86 ± 1.947	.855
WMS-R (SLC)-(f)	49.00 ± 2.115	45.10 ± 2.393	.228
WMS-R (SLC)-(b)	44.97 ± 1.701	44.83 ± 2.169	.960

Data are presented as mean ± standard deviation

*TMT-B* Trail Making Test Part B forward and backwards,* WMS-R* Wechsler Memory Scale-R (memory functions), *L* supine,* S* standing,* SLC* standing with legs-crossed,* f* forward,* b* backwards

*p < .05; **p < .01

**Table 4:** Post hoc* t* tests investigating the significant interaction between position and group on reading time in the Stroop test

Differences	hEDS	HC	*p* value
Stroop L − S	2.07 ± 1.844	− 0.03 ± 1.029	.323
Stroop L − SLC	2.79 ± 1.696	− 2.24 ± 1.132	.017*
Stroop S − SLC	0.72 ± 1.269	− 2.21 ± 1.296	.112

Data are presented as mean ± standard deviation

Different body positioning:* L* supine,* S* standing,* SLC* standing with legs-crossed

*p < . 05

In the context of hEDS, the correlation analysis revealed significant interactions between cognitive performance and comorbid conditions, particularly pain. Higher levels of pain strongly correlated with increased scores on the Winker (|*r*| = 0.584, *p* < 0.001) and COMPASS (|*r*| = 0.584, *p* < 0.001) assessments. Conversely, there were weak negative correlations between pain and cognitive performance across several tasks, including the LPS (|*r*| = − 0.308, *p* = 0.047), Stroop (|*r*| = −0.216, *p* = 0.125), SLC (|*r*| = − 0.279, *p* = 0.048), WMSR (|*r*| = − 0.353, *p* = 0.012), and block-tapping test (|*r*| = − 0.335, *p* = 0.029).

## Discussion

In this pilot study, we found that patients with hEDS exhibited impaired cognitive function compared to HC. Particularly executive function and attentional capacity were impaired while patients with hEDS were in a standing legs-crossed position. Notably, despite patients with hEDS demonstrating higher intelligence than HC, these impairments were evident. This is the first study to demonstrate such detailed cognitive deficits in a homogenous group of patients with hEDS.

Although previous studies with patients with hEDS found no influence of pain or depression on cognitive outcomes [4], our findings suggest a potential link between cognitive function and comorbid conditions. Patients with hEDS reported significantly more autonomic symptoms, anxiety, pain, and daytime sleepiness, as well as poorer mental and physical health than HC, highlighting the profound impact of hEDS on daily functioning and the disabling nature of the condition. Furthermore, 55% of our hEDS cohort had a SFN, suggesting that pain and autonomic dysfunction associated with SFN could potentially contribute to the cognitive difficulties observed in this population. Second, moderate to strong positive correlations were found between reported pain and higher scores on the Winker and COMPASS assessments, indicating an association between pain and increased autonomic and orthostatic symptoms in these patients. Third, there were weak negative correlations between pain and performance on several cognitive tasks, suggesting a possible influence of pain on cognitive impairment. However, despite these findings, the correlations between pain and cognition were not substantial across all cognitive tasks. Previous studies in non-EDS cohorts have demonstrated an impact of pain on cognition [7–9], but similar research in hEDS is limited. One recent study found that pain, fatigue, and depression primarily impacted subjective cognitive complaints, while objective cognitive performance was largely preserved [25]. This may help explain why patients with hEDS often score well on intelligence tests and do not consistently perform worse than HC. Consequently, the extent to which pain influences both autonomic symptoms and cognition in hEDS remains unclear and warrants further investigation.

OI also influenced cognitive performance in patients with hEDS, particularly those diagnosed with POTS. In contrast to HC and patients with POTS, as shown by [13], the legs-crossed standing position impaired executive function in patients with hEDS rather than improving it. Interestingly, in the TMT-B, HC appeared to take longer during the standing cross-legged condition than in patients with hEDS, which contrasts with the Stroop results, where patients with hEDS performed worse in the same posture. Notably, the robust ANOVA did not show an overall effect. Although patients with hEDS showed worse Stroop performance in the standing legs-crossed condition compared to HC, Stroop scores for both groups remained within normative ranges, suggesting that these statistically significant differences may not reflect clinically meaningful impairment and should be interpreted with caution. These findings might suggest that the effects of posture and compensatory maneuvers on executive function may be quite complex. One possible factor is that many patients with hEDS are clinically highly cognitively capable, which may mitigate the attentional demands of leg-crossing. In addition, traits associated with autism spectrum conditions—which are more prevalent in hEDS—may confer strengths in selective attention and executive control, potentially influencing performance on tasks such as the TMT-B. On the other hand, fluctuations in objective cognition might also explain these findings. Thus, these observations highlight both individual variability and the need for caution when interpreting the cognitive effects of postural interventions.

During the task, many patients with hEDS reported difficulty in maintaining the legs-crossed standing posture. Current literature suggests that the standing legs-crossed position can be distracting, as cognitive and motor functions compete for limited attentional resources [47, 48]. In hEDS, this posture may constitute a more complex motor task due to joint hypermobility, instability, and impaired proprioception [49, 50]. The additional motor control and balance demands likely increase attentional load, which can offset or even override the potential cognitive benefits from improved orthostatic perfusion. This may explain why patients with hEDS do not show cognitive improvement in the legs-crossed position, unlike patients with, for example, POTS alone, for whom the maneuver primarily alleviates orthostatic stress without adding substantial motor demands. It might be interesting to include proprioception-specific tests in future studies.

Additionally, the effect of compression garments and physiotherapy on cognition in hEDS could be tested further. Some past studies have shown that compression garments can both relieve pain and improve proprioception [51, 52]. It was also shown that the effect could be increased with additional physiotherapy [52].

The small sample size in this study limits the ability to conduct detailed subgroup analyses or thoroughly explore the relationship between OI and cognition. The washout period would have been too short to fully remove the influence of medications potentially affecting cognition (e.g., stimulants, SNRIs). A potential limitation of our study is the dichotomous reporting of educational level rather than using actual years of education. Given the known impact of educational attainment on cognitive performance, this should be considered when interpreting our results and addressed in future studies. Nonetheless, this is the first study to specifically investigate cognitive performance in patients with hEDS under various conditions, compared to HC. Further studies are needed to better understand these relationships and their clinical implications.

## Conclusion

In this pilot case–control study, patients with hEDS showed indications of impaired cognitive performance compared to HC, particularly in selected domains such as executive function and attention. This occurred despite patients with hEDS displaying a higher intelligence profile. Cognitive performance was closely associated with pain and autonomic symptoms, particularly under orthostatic conditions. Notably, tasks such as standing legs-crossed impaired executive function in patients with hEDS, rather than improving it, as observed in other populations. It must be taken into account that only certain aspects of cognition were assessed in this study, and conclusions should therefore be interpreted with caution. On the basis of our results, future studies should investigate whether interventions such as wearing compression garments and physiotherapy could improve not only proprioception but also cognition. In addition, symptoms such as pain, depression, and fatigue should be treated appropriately to stabilize cognitive functioning. These findings underscore the need for further research into the relationship between orthostatic intolerance, pain, and cognitive function in patients with hEDS.

## Data Availability

Anonymized data and the full trial protocol will be shared by reasonable request from the corresponding author.
